# Derivation and Validation of Generalized Sepsis-induced Acute Respiratory Failure Phenotypes Among Critically Ill Patients: A Retrospective Study

**DOI:** 10.21203/rs.3.rs-4307475/v1

**Published:** 2024-04-30

**Authors:** Tilendra Choudhary, Pulakesh Upadhyaya, Carolyn M. Davis, Philip Yang, Simon Tallowin, Felipe A. Lisboa, Seth A. Schobel, Craig M. Coopersmith, Eric A. Elster, Timothy G. Buchman, Christopher J. Dente, Rishikesan Kamaleswaran

**Affiliations:** Duke University School of Medicine; Duke University School of Medicine; Emory University School of Medicine; Emory University; Uniformed Services University of the Health Sciences; Uniformed Services University of the Health Sciences; Uniformed Services University of the Health Sciences; Emory University School of Medicine; Uniformed Services University of the Health Sciences; Emory University School of Medicine; Emory University School of Medicine; Duke University School of Medicine

**Keywords:** acute respiratory failure, critical care, phenotype, unsupervised machine learning, sepsis-induced ARF

## Abstract

**Background:**

Septic patients who develop acute respiratory failure (ARF) requiring mechanical ventilation represent a heterogenous subgroup of critically ill patients with widely variable clinical characteristics. Identifying distinct phenotypes of these patients may reveal insights about the broader heterogeneity in the clinical course of sepsis. We aimed to derive novel phenotypes of sepsis-induced ARF using observational clinical data and investigate their generalizability across multi-ICU specialties, considering multi-organ dynamics.

**Methods:**

We performed a multi-center retrospective study of ICU patients with sepsis who required mechanical ventilation for ≥24 hours. Data from two different high-volume academic hospital systems were used as a derivation set with N=3,225 medical ICU (MICU) patients and a validation set with N=848 MICU patients. For the multi-ICU validation, we utilized retrospective data from two surgical ICUs at the same hospitals (N=1,577). Clinical data from 24 hours preceding intubation was used to derive distinct phenotypes using an explainable machine learning-based clustering model interpreted by clinical experts.

**Results:**

Four distinct ARF phenotypes were identified: A (severe multi-organ dysfunction (MOD) with a high likelihood of kidney injury and heart failure), B (severe hypoxemic respiratory failure [median P/F=123]), C (mild hypoxia [median P/F=240]), and D (severe MOD with a high likelihood of hepatic injury, coagulopathy, and lactic acidosis). Patients in each phenotype showed differences in clinical course and mortality rates despite similarities in demographics and admission co-morbidities. The phenotypes were reproduced in external validation utilizing an external MICU from second hospital and SICUs from both centers. Kaplan-Meier analysis showed significant difference in 28-day mortality across the phenotypes (p<0.01) and consistent across both centers. The phenotypes demonstrated differences in treatment effects associated with high positive end-expiratory pressure (PEEP) strategy.

**Conclusion:**

The phenotypes demonstrated unique patterns of organ injury and differences in clinical outcomes, which may help inform future research and clinical trial design for tailored management strategies.

## Introduction

Sepsis is a heterogeneous syndrome that is characterized by life-threatening organ dysfunction due to a dysregulated host response to infection [1]. Despite advances in our knowledge and improvements in management strategies, sepsis continues to be one of the leading causes of death worldwide and remains a serious medical emergency [2, 3]. Patients with sepsis who develop acute respiratory failure (ARF) requiring mechanical ventilation represent a unique and complex subgroup [4–6]. ARF is one of the most common complications in sepsis and one of the strongest risk factors for mortality. This subgroup of patients also has heterogeneous risk factors, etiologies, pathophysiology, and immunopathogenic responses that contribute to ARF, as well as divergent clinical courses and outcomes [7]. A typical manifestation of ARF in patients with sepsis is the acute respiratory distress syndrome (ARDS). In addition to ARDS, these patients also frequently develop other extra-pulmonary organ dysfunctions that lead to increased complications and high mortality.

Clinically recognizable phenomena that are widely observed in sepsis and ARF, such as vital sign abnormalities (dyspnea, hypotension, tachypnea, oxygen desaturation, etc.) and laboratory abnormalities (lactic acidosis, hypoxemia and/or hypercapnia on arterial blood gas, etc.), are only superficial representations of complex pathophysiological and environmental interactions. Furthermore, they do not provide specific information regarding the heterogeneous clinical trajectories and outcomes, which contribute to the ongoing challenges in developing targeted management strategies and improving outcomes in sepsis-induced ARF. However, there may be subtle patterns of physiologic data and clinical features that may be unclear to clinicians at the bedside but are uncovered with the aid of machine learning (ML) models. The ML models can help recognize these patterns as unique phenotypes within heterogeneous syndromes such as sepsis-induced ARF. While latent class analysis (LCA) techniques have been used to derive hyper- and hypo-inflammatory phenotypes in ARDS with potential differences in treatment responses, these phenotypes only apply to a specific subset of patients with a key manifestation of ARF. In addition to ARDS, there is a need to investigate other organ dysfunctions that are related to sepsis-induced ARF. Moreover, there is a need to identify generalizable phenotypes among patients with sepsis to elucidate possible mechanisms of complex clinical courses and targetable features pertaining to certain phenotypes.

We sought to utilize pre-intubation clinical data to develop generalizable phenotypes that would correlate with not only the risk of developing ARDS as an endpoint, but also to investigate more complex multi-organ failure trajectories observed within this cohort [8]. We further sought to compare the derived generalized sepsis-induced ARF phenotypes against the binarized hyper- and hypo-inflammatory subphenotypes to characterize potential overlaps between these approaches. To understand the latent phenotypes in critically ill patients with sepsis-induced ARF, we developed a multi-phased unsupervised ML model to systematically identify novel phenotypes using multi-variable data collected from electronic medical records (EMR).

## Methods

### Study Design

This is a multi-center retrospective cohort study conducted at two high volume academic hospitals located in the southeastern United States. Adult patients (≥ 18 years) admitted to the medical or surgical intensive care units (MICU or SICU) at either of these two metropolitan academic hospitals, Emory University Hospital and Grady Memorial Hospital (Atlanta, GA) with sepsis [based on the sepsis-3 criteria [1]] between 2016–2021 and developed ARF during their hospital admission were included [9]. Emory is a quaternary care hospital specializing in the care of adult critically ill patients, whereas Grady is known as a safety-net hospital. ARF was defined as requiring ≥ 24 hours of invasive mechanical ventilation (IMV) for medical ICU patients. For surgical ICU patients, it is defined as ≥ 24 hours of IMV even after 48 hours from surgery.

### Participants

IMV patients included in our cohort were adult patients admitted to the hospital who were diagnosed with sepsis and during their hospital course required mechanical ventilation as well as adult surgical patients whose post-operative course was complicated, requiring *post-surgical IMV* lasting at least 24 hours. Here, *post-surgical IMV* refers to initial IMV, re-ventilation or remaining in IMV state after 48th hour from the surgery completion. Due to our interests in identifying the phenotypes early in the course of sepsis-induced ARF, we utilized up to 24 hours of data preceding the time of index IMV (or *post-surgical IMV*) in the study. Data collected from the EMR including laboratory values and vital signs, were used for phenotyping. A complete list of these clinical factors or features can be found in Table E1 in the online data supplement. All factors represent clinical values that were routinely collected and recorded in the EMR. A set of demographic variables (e.g., age, sex, race, ethnicity), mortality, and comorbidity information were also included for further analysis of derived phenotypes. We created two separate datasets corresponding to MICU and SICU patients. We defined index time of IMV in the MICU dataset as the time at which the first mechanical ventilation parameters (positive end-expiratory pressure (PEEP), tidal volume, and/or plateau pressure) were recorded in the EMR in patients who met the above inclusion criteria; while for SICU dataset, it is the time of first ventilation parameters recorded from 48-hour mark after the surgery.

**Figure 1:**
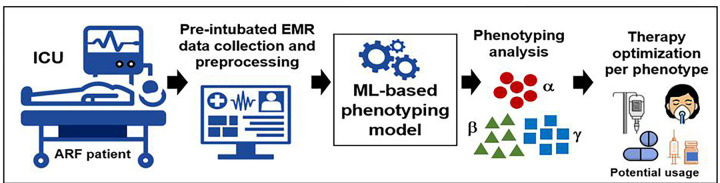
Overall study pipeline of our ARF phenotyping approach showing data extraction, preprocessing, model development for clustering, phenotype analysis and potential usage.

We excluded patients who did not meet sepsis-3 criteria, patients admitted to neurological ICUs, patients admitted to the ICU post-operatively who were ventilated only for ≤ 24 hours after 48 hours from their surgeries, or those whose EMR data did not include any hourly collected physiological data up to 24 hours prior to IMV. To derive enriched phenotypes for sepsis-induced ARF, we developed a high-fidelity unsupervised ML-based approach that incorporates a broad set of routinely collected clinical variables. The overall study pipeline is shown in Fig. 1.

### Procedure

We adopted a multi-center derivation and validation study design by first deriving phenotypes using medical ICU (MICU) data from Emory University Hospital, and then validating this phenotyping algorithm against MICU data collected from Grady Memorial Hospital. The phenotyping was further validated with surgical ICU (SICU) data from both the hospitals. The two hospitals serve unique and diverse patient populations located within the metropolitan southeast United States. Data used to derive and validate our algorithm were collected from the same years across the two hospitals.

We applied median aggregation to the data of our cohort, across the pre-ventilation time-window, for all routinely collected clinical features. We dropped features having more than 85% of missing values in the aggregated data. Subsequently, we used multivariate imputation by chained equations (MICE) algorithm on the training data [10] to impute missing data and correct outliers. Finally, a total of 50 robust and independent clinical features were selected after a Pearson’s correlation analysis, also listed in the online data supplemental Table E1. We then normalized the data and used Uniform Manifold Approximation and Projection (UMAP) method to reduce dimensionality of the multivariate dataset and project onto a two-dimensions [11]. Finally, we used a *k*-means (centroid-based) clustering algorithm that yielded four clusters. The derived clusters were analyzed for their most important features using SHapley Additive exPlanations (SHAP) values [12]. The derived phenotypes were then examined and interpreted by physicians P.Y., C.M.D. and C.M.C.

#### Validation of the Phenotypes in External Dataset

We trained a logistic regression (LR) ML model for phenotype prediction on the derivation data with high accuracy. This model outperformed other ML models such as random forests, support vector machines (SVM) and Gaussian Naïve Bayes classifier (see Figures E4 and E5 in the online data supplement). Finally, we applied this trained classifier model to the validation datasets. The purpose was to evaluate the robustness of the classifier in identifying similar phenotypes in ‘unseen’ and external data from a different medical center.

#### Validation of the Sepsis-induced ARF Phenotypes Against Hyper/Hypo Inflammatory ARDS Subtypes

To investigate how the contributed phenotypes compare to the existing work in the field, we sought to compare the proposed sepsis-induced ARF phenotypes to the ARDS hyper- and hypo-inflammatory subtypes. Calfee and colleagues investigated clinical and biological data to elucidate two phenotypes in various ARDS cohorts, namely hyper- and hypoinflammatory [13–15]. In a recent extension of these approaches, Sinha et al. proposed a clinical only-model that provided robust discrimination of the ARDS cohort into the two relevant subtypes [13]. However, due to the unavailability of the pre-trained models and labeled data contributed by those groups, we were unable to directly evaluate the classifier against our own data, thus we developed a pragmatic alternative, which utilized the value-intervals of their class-defining features for further heuristic characterization.

### Statistical Analysis

Statistical analyses were performed using python libraries. Patient characteristics and endotype factors that represent continuous variables were analyzed using a Kruskal-Wallis test. Categorical variables were analyzed using a Chi-squared test. A multivariate log rank test was performed when comparing multiple variables and a p-value of ≤ 0.05 was used for statistical significance. We report the specific statistical dichotomization of the hyper and hypo-inflammatory subtypes based on the ranges of each variable as provided by Sinha et al. in the supplemental documents S2-S6 [13, 16].

## Results

### Patient Characteristics:

In this retrospective study, a total of 3349 encounters from 3225 unique patients admitted to MICU at Emory University Hospital (Atlanta, GA) were selected from the derivation data for initial phenotyping. The cohort in our study consists of patients across a wide range of demographic variables, such as age (mean: 62.3 ± 15.3 years), sex (male: 53.1%), and race (Caucasian: 42.2%, African American: 47.4%). For validating our phenotyping algorithm, 867 encounters from N = 848 unique and diverse patients were selected from the MICU of Grady Memorial Hospital (Atlanta, GA). Characteristics of these cohorts are described in Tables 1 and 2. For validation of multi-ICU generalization, we used SICU patients from Emory [1128 encounters (N = 1112)] and Grady [466 encounters (N = 465)] hospitals, who required intubation even after 48 hours of completion of their surgeries. Characteristics of these SICU patients are available in the supplemental Tables E2 and E3.

### Phenotyping Results:

**Figure 2:**
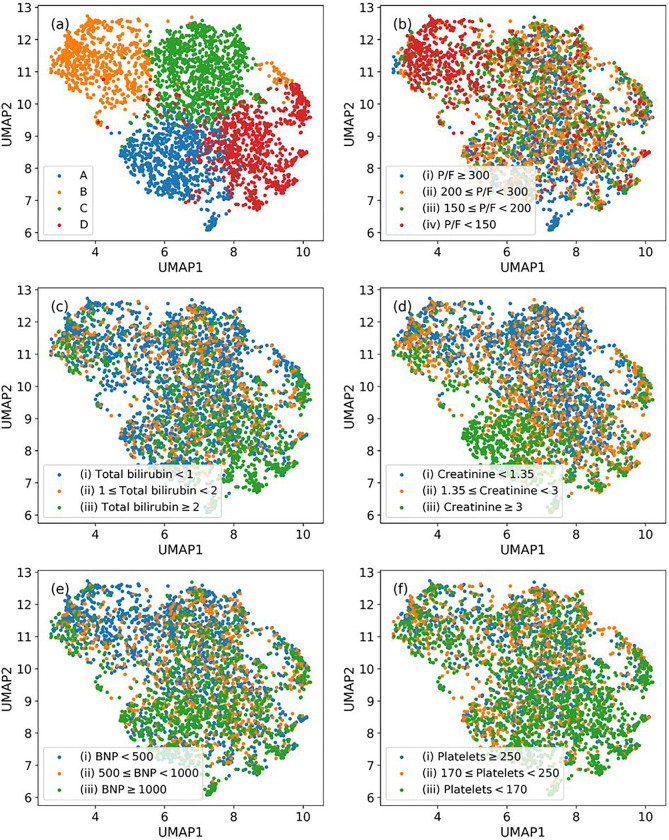
UMAP projections showing distribution of the derivation data for ARF phenotyping and feature variations. (a) UMAP representing all ARF phenotypes, and (b)-(f) UMAP representations showing variabilities in P/F ratio (mmHg), bilirubin total (mg/dL), creatinine (mg/dL), BNP (pg/mL), and platelets (×10^3^/μL), respectively.

Our clustering algorithm for deriving enriched ARF phenotypes characterized by various risks profiles yielded four clusters on the derivation data with a silhouette score of 0.418, Calinksi-Harabasz score (variance ratio criterion) of 3555.87, and Davies-Bouldin score of 0.79. The optimal number of clusters was decided by achieving a combination of highest silhouette score, highest Calinksi-Harabasz score and lowest Davies-Bouldin score for the clustering. The patient distributions are shown in Figure 2 using a 2-D Uniform Manifold Approximation and Projection (UMAP) representing formed clusters and variations of important clinical features across these distributions. SHAP values were used to identify the important features that distinguished one cluster from another. SHAP plots are available in Figure E3 in the online data supplement. Subsequently, critical care physician experts helped interpret and characterize the four clusters as phenotypes based on their characteristics.

Table 1 summarizes the clinical and demographic variables for each of the four derived ARF phenotypes along with their mortality outcomes. The first phenotype (N=825 patients) has ARF patients with multiple laboratory abnormalities, such as highest median levels of creatinine (median: 3.47, IQR: 1.89–5.74 mg/dL), blood urea nitrogen (BUN) (median: 56, IQR: 34–80.25 mg/dL) and B-type natriuretic peptide (BNP) (median: 750.5, IQR: 251.25–1775.5 pg/mL). Based on the characteristics, we named this phenotype A (MOD-1) with *severe multiple organ dysfunction (MOD) showing a high likelihood of kidney injury and heart failure*. The second phenotype (N=689 patients) consists of patients with severe hypoxia and clinical characteristics suggestive of non-radiographic features of severe ARDS (low partial pressure of oxygen (PaO_2_) to fraction of inspired oxygen (FiO_2_) ratio [P/F ratio] [median: 123, IQR: 90–185 mmHg] and high FiO_2_ [mean: 0.8, IQR: 0.6–1]) and has the highest mortality (51%). We called it phenotype B (*severe hypoxemic respiratory failure*). The third phenotype (N=959 patients) consists of patients with no evidence of organ failure other than mild hypoxia (median P/F ratio: 240 [95% CI: 185, 317.7]) and normal lactic acid levels (median: 1.42 mmol/L). We called it phenotype C (*mild hypoxia*). The fourth phenotype (N=806) consists of ARF patients with highest total bilirubin (median: 1.2, IQR: 0.6–4.1 mg/dL) and highest D-dimer levels, lowest platelets (median: 118, IQR: 53–202.8 ×10^3^/μL) and highest lactic acid (median: 2.35, IQR: 1.43–4.67 mmol/L) suggesting multi-system organ dysfunction. As such, we named this phenotype D (MOD-2) with *severe MOD showing a high likelihood of hepatic injury, coagulopathy and lactic acidosis*. From Table 1, we observed that phenotype B has the highest mortality (51%), followed by phenotype D (49.6%) and phenotype A (40.9%). The relatively healthier phenotype B had a mortality of 21.4%. Phenotype D is also characterized by the highest proportion of patients with septic shock (n=651 patient encounters (79.5%), whereas C consists of the lowest proportion of septic-shock patients (450 encounters, 45.3%). Thus, our phenotypes not only identified distinct patterns of organ injury in patients with sepsis-induced ARF, but also different rates of mortality and septic-shock distributions. To confirm more insights of MOD profiles in phenotypes, a set of all six individual SOFA were analyzed from the pre-intubation window for each phenotype. The maximum method was used for their aggregation. Supplemental Table E4 presents SOFA score-maps for Emory MICU data, where the findings clearly align with our phenotype characterizations.

**Figure 3:**
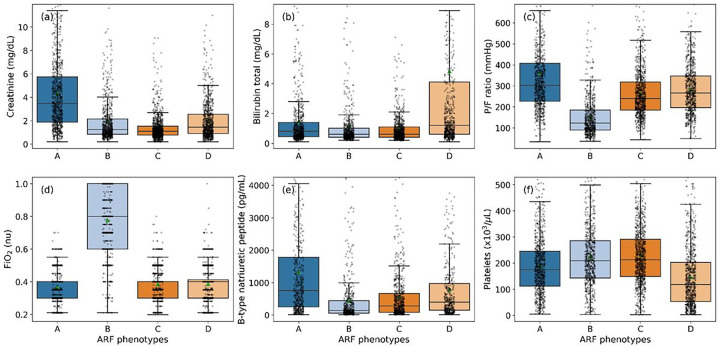
Visualization of feature variations across different ARF phenotypes of the derivation data. (a)-(f) boxplot representations of different phenotypes showing variabilities in creatinine (mg/dL), bilirubin total (mg/dL), P/F ratio (mmHg), FiO_2_, BNP (pg/mL), and platelets (×10^3^/μL), respectively to show fitness of individual organs. Presented phenotyping results were significant (p<0.001, Kruskal-Wallis test) for each of the variables.

Boxplots were drawn to illustrate the variabilities in certain prominent features such as creatinine (renal), total bilirubin (hepatic), P/F ratio and FiO_2_ (respiratory), BNP (cardiac), and platelets (coagulopathy), as shown in Figure 3(a)-(f). We also calculated the age-adjusted Charlson Comorbidity Index based on admission diagnosis ICD-9 codes for all four phenotypes, and they are 2.3 (95% CI: 2.21, 2.4), 1.94 (95% CI: 1.84, 2.05), 1.99 (95% CI: 1.9, 2.07) and 2.06 (95% CI: 1.97, 2.16), respectively.

### External and Multi-specialty Validation of Sepsis-induced ARF Phenotypes:

To validate our phenotyping algorithm, we utilized an external hospital’s MICU cohort from Grady Memorial Hospital, Atlanta, GA. Our methodology involves training a supervised learning (logistic regression) classifier on the derivation dataset to predict the corresponding phenotype. Thereafter, we employed the trained model on the validation dataset to determine the phenotype for each patient encounter. We summarize the phenotype validation results in Table 2. These results indicate that most features across the four phenotypes remain consistent in the validation dataset, highlighting the reliability and generalizability of our phenotyping approach.

For further analysis of the phenotypes, Figure E1 in the online data supplement shows radar diagrams illustrating average variations of all clinical feature values across four formed phenotypes of the derivation and validation data, where all features are normalized in the range 0–1. Additionally, radar diagrams in Figure E2 in the online data supplement presents distributions of demographic variables and mortality outcomes across various phenotypes of the derivation and validation data. Additionally, our phenotyping approach was also validated on SICU cohorts of both Emory and Grady hospitals. Their phenotyping results are listed in the supplemental Tables E2 and E3. We also analyzed aggregated pre-intubated individual SOFA for these datasets, and the results are listed in Supplemental Tables E5-E7. They show consistency in earlier results obtained from the derivation data.

### Short-term Survival Analysis:

**Figure 4:**
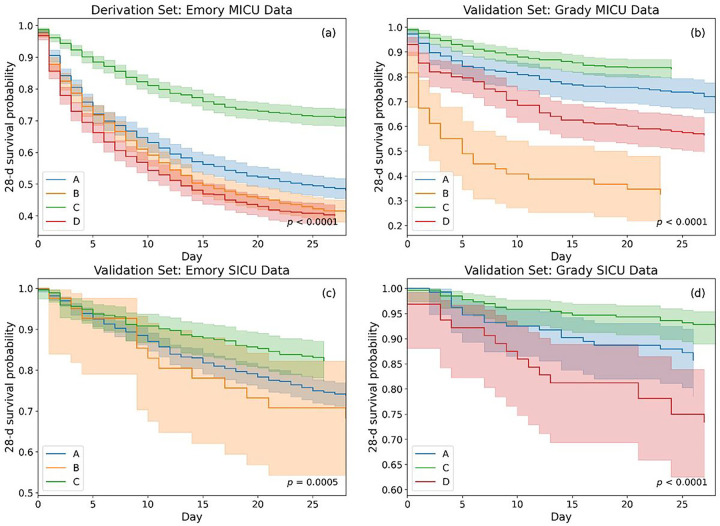
Kaplan-Meier curve showing 28-day survival rates for ARF patients stratified by phenotypes developed for (a) derivation set: Emory MICU data, and (b) validation set: Grady MICU data, (c) validation set: Emory SICU data, and (d) validation set: Grady SICU data. Survival was analyzed from the time of intubation. Survival probabilities (solid line) with their confidence intervals (faded region) are represented for different phenotypes via different color-codes.

Trajectory of short-term outcomes can provide a better differentiation among phenotypes. For a 28-d short-term analysis, average vent-free days (VFD) were found as 10.4, 8.6, 15.4 and 8.5, respectively for phenotypes A to D of the derivation set. To evaluate the survival probability of patients in each phenotype, we plotted Kaplan-Meier curves [17] for a 28-day period following intubation, as shown in Figure 4. The analysis was performed for derivation and validation datasets, where survival traces of phenotype D of Emory SICU (N=12) and B of Grady SICU (N=5) were omitted here due to having their small sample sizes. We observed that the mortality trends across various phenotypes were consistent for MICU and SICU of both centers (*p*-value for trend < 0.001), with phenotype C having the best survival followed by A, and phenotypes B and D having the poor survival rates in both centers. This suggests that our phenotyping approach is generalizable in identifying the least and the most critical phenotypes in terms of short-term survival for ARF patients with different demographic characteristics.

### Exploratory Analyses of Clinical Differences among the Phenotypes:

We also performed exploratory analyses to examine whether the phenotypes would demonstrate different outcomes or clinical patterns in relation to high PEEP (PEEP ≥ 10) treatments. Within phenotypes, 16.7% in A, 49.6% in B, 24% in C, and 16.2% in D were administered with PEEP ≥ 10 regime on mechanical ventilator. We conducted an analysis to estimate the effects of high PEEP (PEEP ≥ 10) using a propensity score matching scheme on 28-day short-term mortality, by considering lab-values and demographics as confounding variables. Average treatment effects (ATE) with 95% confidence intervals were obtained as 0.04 (−0.08, 0.16) for A, −0.05 (−0.13, 0.02) for B, 0.07 (−0.01, 0.15) for C, and −0.07 (−0.19, 0.05) for D. A negative ATE suggests reduced mortality outcomes for the treated group. We also plotted Kaplan-Meier curves between patients who received high PEEP and those who did not within each of the phenotypes.

**Figure 5:**
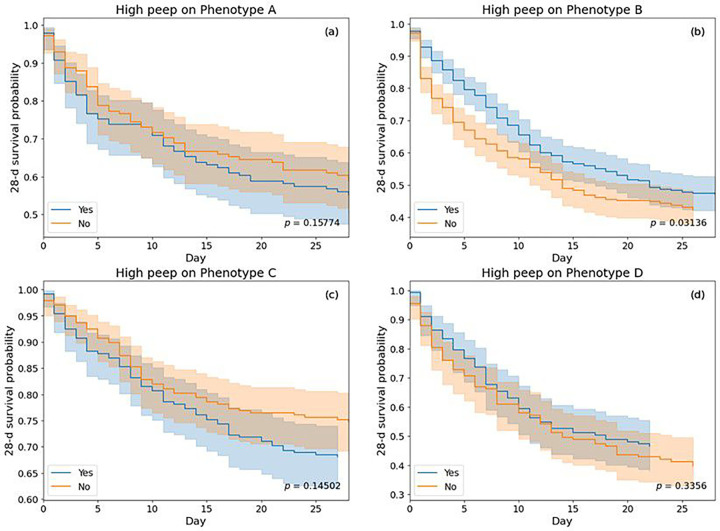
Kaplan-Meier survival curves showing treatment effects of high PEEP (PEEP≥10) and low PEEP (<10) regimes on propensity matched ARF phenotypes from the derivation dataset. Survival probabilities (solid line) with their confidence intervals (faded region) are represented for treated and untreated cohorts within each phenotype.

In phenotype B with severe hypoxic respiratory failure, higher PEEP (≥10) was associated with significantly better survival than lower PEEP (<10), but the opposite association was seen in phenotype C. Among both MOD phenotypes, higher PEEP was found effective for D, whereas it was ineffective for A. However, the PEEP strategy was not significantly associated with survival in both these phenotypes (Figure 5). We must emphasize that this analysis was purely exploratory in nature and was carried out to examine the feasibility of further research on the treatment effects of various therapies.

### Validation of the phenotypes against the Hyper/Hypo Inflammatory Phenotypes:

We further sought to investigate how the phenotypes derived from the results above compared to the binarized phenotypes, namely the hyperinflammatory and hypoinflammatory phenotypes.[13,14] By comparing the clinical values reported, our results suggested that patients in phenotype A (MOD-1) and D (MOD-2) were most likely associated with hyperinflammation characterized by high values of total bilirubin (mean A:1.4, D:4.8 mg/dL) and creatinine (mean A:4.3, D:2 mg/dL), and low values of platelet count (mean A:191, D:147 ×10^3^/μL), bicarbonate (mean A:22.4, D:20.7 mmol/L), PaCO_2_ (mean A:38.6, D:34.2 mmHg) and hemoglobin (mean A:9.2, D:9 g/dL), which were consistent with the values of these markers in the hyperinflammatory subphenotype from the previous works [13,14]. Phenotype B with severe hypoxemic respiratory failure demonstrated features that were consistent with neither hyper- nor hypo-inflammatory phenotype, suggesting that this phenotype could either consist of a mix of both phenotypes or represent a completely novel phenotype. On the contrary, patients in C were associated with hypoinflammatory characteristics with lowest values of total bilirubin (mean:1.1 mg/dL) and creatinine (mean:1.4 mg/dL), and highest values of platelet count (mean:231×10^3^/μL), bicarbonate (mean:26 mmol/L), PaCO_2_ (mean:42.5 mmHg) and hemoglobin (mean:11.6 g/dL). In comparison to the earlier works, the patient population and variables included were not the same. For example, we did not use biologically derived features such as interleukin-6/8 and intercellular adhesion molecule 1. Hence, this characterization of hyper/hypo-inflammatory subgroups in our identified sepsis- induced ARF phenotypes needs further investigation.

### Practice Variance During COVID-19:

When evaluating the derivation strategy independently during the 2020–2021 data, we found that they were consistent with that of pre-COVID-19 years, without significant variance in the distribution of the phenotypes. Relevant details on the sensitivity analyses are available in Figure E6 in the online data supplement.

## Discussion

In this study, we performed UMAP projection and unsupervised clustering to derive novel phenotypes of sepsis-induced ARF. The phenotypes derived using early clinical data from the pre-intubation phase of sepsis-induced ARF not only demonstrated unique patterns of organ injury, but also correlated with differences in mortality and exhibited potential differences in outcomes in relation to High vs. Low PEEP strategy. Furthermore, the characteristics of the phenotypes remained consistent in the validation datasets. The performance was evaluated on comprehensive datasets including rich and diverse cohorts of patients with varying comorbidities across different demographics.

Sepsis and ARF are both heterogeneous syndromes with diverse risk factors, etiologies, clinical presentations, prognosis, pathophysiology, and immune response mechanisms that continue to pose limitations for improving outcomes. The present study is unique in that it identified novel phenotypes in a broader population of patients with sepsis-induced ARF, and provides valuable information about their distinct clinical characteristics, outcomes, and potential differences in treatment responses. Prior studies have tried to address this issue by deriving phenotypes separately in sepsis and in ARDS, but have not focused on phenotypes of sepsis-induced ARF[15, 18]. For example, Aliberti *et al. *described four phenotypes of patients with community-acquired pneumonia (CAP) in presence of ARF or severe sepsis (SS): CAP without ARF or SS, CAP with ARF only, CAP with SS only and CAP with both ARF and SS [19]. Essay *et al.* presented an algorithm for phenotyping ARF patients using remotely monitored ICU (tele-ICU) data from more than 200 patients and validated it using a large cohort EMR data from 46 ICUs in southwest United States [20]. The validation was done by comparing the output of the phenotyping algorithm to a manual review, and the common causes of misclassification were noted [21]. Unlike our work, this study does not characterize various degrees of MOD associated with ARF and the severity of such outcomes. It only characterizes patients based on the sequence of different respiratory support they received. The set of features analyzed is also limited; in contrast, this work considers an expanded set of clinical features for phenotyping. While many of these studies identify phenotypes in sepsis at large [22–26], they have not examined phenotypes that may exist specifically within sepsis-induced ARF. Similarly, LCA-derived phenotypes of ARDS have been well-studied, but do not directly apply to ventilated patients with sepsis who do not satisfy the Berlin definition of ARDS.

Although previously studies have highlighted the limitations in using EMR for phenotyping such as complex, inaccurate, and missing data problems [27, 28], our approach uses a wide range of clinical features from EMR data and has been shown to work efficiently in both derivation and validation datasets. Our study phenotypes show results consistent with the corresponding MODs. For example, the high mortality of phenotype B (severe hypoxic respiratory failure) is 51%, which is close to the numbers reported (34–46%) in previous studies [3]. Our phenotyping algorithm from EMR data with high richness and freedom from bias, have been shown to be generalizable and consistent across multiple patient groups from different hospitals, also suggested by prior studies [29, 30].

The unique clinical characteristics of the derived phenotypes and the results of our exploratory analyses are highly informative and can be hypothesis-generating for future research. For example, phenotype A and D appear to suffer from multi-system organ failure (with differences in the organs involved), and may require tailored interventions according to their specific patterns of organ injury. Our results also showed that patients in phenotype A and D were likely to exhibit hyperinflammatory characteristics. However, ARF patients in A had better survival than patients in D overall (Fig. 4). Notably, those who received PEEP ≥ 10 had much better survival than those who did not in phenotype D, but this difference in survival was not better in phenotype A, further suggesting potential differences between the two MOD phenotypes. Phenotype B (severe hypoxemic respiratory failure) is characterized by the most severe degree of hypoxia and associated with the highest mortality. Higher PEEP strategy was also associated with better survival in phenotype B. This could represent the phenotype in which adjunctive treatments for ARDS and severe hypoxemia are needed most often and may be useful for predictive and prognostic enrichment in future clinical trials for ARDS and severe hypoxic respiratory failure. Lastly, phenotype C (mild hypoxia) represents the phenotype with only modest degree of hypoxemia, no other organ injury, and the best outcomes. It was also observed that patients in phenotype C were likely to be hypoinflammatory. We propose that these differences between the phenotypes are worthy of further investigation in future validation studies and clinical trials, and have the potential to provide valuable information regarding potential complications and prognosis, in addition to aid in developing tailored management strategies. Furthermore, the present study phenotypes were identified using early clinical data from the pre-intubation phase of sepsis-induced ARF, which may facilitate prompt classification of patients and candidate selection in future research, as well as timely implementation of tailored management strategies when applied to real clinical settings.

The study has several limitations. First, only routinely collected clinical features in the EMR were used to derive the phenotypes, and integration with other data such as protein biomarkers, clinicians’ impression, immune cell expression or pathogen features during derivation could change assignments of ARF phenotypes. Second, our method relies on the analysis of 24-hour pre-intubation retrospective data, a population aggregation window based on the median for each feature. Third, as the missingness in data was common for some features included in the phenotyping model, MICE-based multivariate imputation strategy was used in the initial analysis. However, features with high missing values were excluded. Fourth, clinical phenotypes were identified and characterized from a single high-volume integrated health system in the USA with MICU patients. However, a large range of data collection years was considered. Although obtained phenotypes were observed to be generalizable in other hospital system data examined across multiple ICUs, further exploration and extensive validation are required, especially using data from randomized clinical trials, prospective studies, low- and middle-income countries, and longitudinal cohorts.

Our proposed model with explainable artificial intelligence (AI) has an ability to identify important features out of all clinical variables and lab values, which need to be focused specifically to group sepsis-induced ARF patients. The strengths of this study include the usage of large comprehensive datasets from multiple hospitals across multiple ICU, derivation and validation of phenotypes with different hospitals’ data, inclusion of a broad set of routinely collected clinical variables, and mapping of MOD, ARF severity, and mortality outcomes. The consistency in characteristics of the phenotypes in the validation datasets supports the generalizability and reproducibility of our results. Thus, the proposed derivation pipeline for patients with sepsis-induced ARF was found helpful in identifying unique and potentially unclear patterns as well as patient characteristics that can then be utilized both for clinical management and for future research.

In conclusion, we have derived novel phenotypes of sepsis-induced ARF with distinct clinical characteristics and outcomes. The phenotypes are associated with distinct patterns of organ injury, such as cardiac/renal dysfunction, hepatic dysfunction and coagulopathy, and severe hypoxemic respiratory failure resembling ARDS. The phenotypes also demonstrated potential differences in treatment responses to common clinical interventions for sepsis and ARF. Our method can offer valuable knowledge into the diversity of ARF patients with regard to their clinical presentation, prognosis and likelihood of additional complications, which may have a significant impact on the development of tailored management strategies, discussions about goals of care, and patient selection for future clinical trials.

## Figures and Tables

**Table 1:** Summary of patient characteristics of the derivation cohort (Emory MICU) and its phenotypes.

Parameters	Whole cohort	A	B	C	D	*p*-value
**count (%)**	3349 (100)	845 (25.2)	692 (20.7)	993 (29.7)	819 (24.4)	-
**Mortality** *	1295, 40.2%	337, 40.9%	353, 51.2%	205, 21.4%	400, 49.6%	-
**Age, mean(std)**	62.3 (15.5)	64.8 (14.6)	62.0 (15.0)	61.7 (16.7)	60.8 (15.0)	<0.001
**Males, count (%)**	1814 (54.2)	492 (58.2)	363 (52.5)	547 (55.1)	412 (50.3)	-
**Race: African American or Black, count (%)**	1624 (48.5)	457 (54.1)	337 (48.7)	469 (47.2)	361 (44.1)	< 0.001
**Race: Caucasian or White, count (%)**	1442 (43.1)	341 (40.4)	280 (40.5)	453 (45.6)	368 (44.9)	
**Ethnicity: Hispanic, count (%)**	129 (3.9)	23 (2.7)	38 (5.5)	30 (3.0)	38 (4.6)	<0.001
**Ethnicity: Non-Hispanic, count (%)**	2975 (88.8)	770 (91.1)	589 (85.1)	903 (90.9)	713 (87.1)	
**P/F ratio, m(IQR)**	240.0 [162.0,334.9]	302.3 [226.7,406.4]	123.3 [90.0,185.0]	240.0 [185.0,317.7]	266.5 [196.5,346.7]	<0.001
**S/F ratio, m(lQR)**	245.0 [188.5,315.6]	250.0 [240.0,326.7]	120.8 [97.5,154.8]	248.8 [232.5,320.4]	247.5 [220.0,325.0]	<0.001
**FiO_2_, m(IQR)**	0.4 [0.3,0.5]	0.4 [0.3,0.4]	0.8 [0.6,1.0]	0.4 [0.3,0.4]	0.4 [0.3,0.4]	<0.001
**PaO_2_, m(IQR)**	94.0 [77.0,124.0]	107.0 [86.0,146.1]	81.0 [67.0,102.0]	92.0 [77.7,116.1]	99.2 [79.6,125.9]	<0.001
**PaCO_2_, m(IQR)**	37.0 [32.0,43.0]	37.0 [32.0,43.0]	38.5 [33.0,45.0]	39.0 [34.0,46.9]	33.0 [29.0,38.0]	<0.001
**MAP, m(IQR)**	82.0 [75.0,90.2]	79.0 [73.0,87.5]	85.0 [77.0,94.0]	87.0 [80.0,95.0]	77.0 [72.0,84.0]	<0.001
**Creatinine, m(IQR)**	1.4 [0.9,2.9]	3.5 [1.9,5.7]	1.3 [0.8,2.1]	1.1 [0.8,1.5]	1.4 [0.9,2.5]	<0.001
**Bilirubin total, m(IQR)**	0.7 [0.5,1.4]	0.8 [0.5,1.4]	0.6 [0.4,1.0]	0.6 [0.4,1.1]	1.2 [0.6,4.1]	<0.001
**Albumin, m(IQR)**	3.0 [2.6,3.5]	2.9 [2.5,3.3]	3.1 [2.7,3.5]	3.4 [3.0,3.8]	2.6 [2.2,2.9]	<0.001
**Lactic acid, m(IQR)**	1.6 [1.2,2.6]	1.5 [1.2,2.1]	1.6 [1.2,2.3]	1.4 [1.1,2.0]	2.3 [1.4,4.7]	<0.001
**D-dimer, m(IQR)**	2678.2 [1141.2,9258.5]	3177.0 [1427.0,6981.5]	1573.0 [979.0,4622.0]	1686.5 [897.8,5183.0]	9828.0 [3187.0,27545.0]	<0.001
**Platelets, m(IQR)**	184.0 [110.2,265.8]	175.0 [112.0,245.0]	209.0 [143.0,286.0]	213.0 [149.0,291.0]	118.0 [53.0,202.8]	<0.001
**Hemoglobin, m(IQR)**	9.8 [8.2,12.0]	8.8 [7.8,10.3]	11.0 [9.0,12.8]	11.5 [9.7,13.4]	8.6 [7.7,10.2]	<0.001
**BNP, m(IQR)**	328.0 [100.0,915.0]	750.5 [251.2,1775.5]	142.8 [60.0,441.8]	279.0 [82.0,664.0]	396.0 [147.5,966.0]	<0.001
**BUN, m(IQR)**	30.0 [19.0,50.0]	56.0 [34.0,80.2]	28.0 [17.5,46.0]	22.0 [14.0,33.0]	28.0 [18.0,42.0]	<0.001
**SOFA max total, m(IQR)**	7.0 [4.0,9.0]	8.0 [5.0,10.0]	6.0 [4.0,8.0]	5.0 [3.0,7.0]	8.0 [6.0,11.0]	<0.001
**GCS total score, m(IQR)**	14.0 [12.0,15.0]	14.0 [12.0,15.0]	15.0 [11.2,15.0]	14.0 [12.0,15.0]	14.0 [11.0,15.0]	<0.001
**PEEP, m(IQR)**	6.0 [6.0,10.0]	6.0 [6.0,8.0]	8.0 [6.0,11.0]	6.0 [6.0,8.0]	6.0 [6.0,8.0]	<0.001

For clinical variables, this table lists the medians and interquartile ranges (IQR: Q1-Q2) for each phenotype as well as for the whole cohort. The p-value is also provided for each variable to indicate the statistical significance of the differences among the phenotypes. For evaluating statistical significance, Kruskal-Wallis test was performed for continuous variables and Chi-squared test was used for categorical variables.

* Mortality was computed with respect to patients (not encounters).

**Abbreviations used –** count: total encounters, mean: average, std: standard deviation, m: median, IQR: interquartile range, PaO_2_: partial pressure of oxygen, SpO_2_: peripheral oxygen saturation level, FiO_2_: fraction of inspired oxygen, P/F: PaO_2_/FiO_2_ ratio, S/F: SpO_2_/FiO_2_ ratio, PaCO_2_: partial pressure of carbon dioxide in arterial blood, MAP: mean arterial blood pressure, Resp.: respiration, BNP: B-type natriuretic peptide, BUN: blood urea nitrogen, SOFA: sequential organ failure assessment, GCS: Glasgow coma scale.

**Measurement units –** P/F ratio, PaO2, PaCO2, and MAP: mmHg; S/F ratio and FiO_2_: unitless; creatinine and bilirubin total: mg/dL; albumin: g/L; lactic acid: mmol/L; D-dimer: ng/mL; platelets: ×10^3^/μL; hemoglobin: g/dL; BNP: pg/mL; BUN: mg/dL.

**Table 2:** Summary of patient characteristics of the validation cohort (Grady MICU) and its phenotypes.

Parameters	Whole cohort	A	B	C	D	*p*-value
**count (%)**	867 (100)	214 (24.7)	49 (5.7)	404 (46.6)	200 (23.1)	-
**Mortality** *	294, 34.67%	80, 38.28%	34, 69.39%	83, 20.75%	97, 48.5%	-
**Age, mean(std)**	59.6 (15.1)	61.6 (13.9)	64.2 (13.1)	59.0 (16.1)	57.7 (14.3)	0.007
**Males, count (%)**	531 (61.2)	128 (59.8)	26 (53.1)	257 (63.6)	120 (60.0)	-
**Race: African American or Black, count (%)**	670 (77.3)	171 (79.9)	35 (71.4)	307 (76.0)	157 (78.5)	0.273
**Race: Caucasian or White, count (%)**	126 (14.5)	22 (10.3)	9 (18.4)	69 (17.1)	26 (13.0)	
**Ethnicity: Hispanic, count (%)**	39 (4.5)	18 (8.4)	2 (4.1)	9 (2.2)	10 (5.0)	0.029
**Ethnicity: Non-Hispanic, count (%)**	818 (94.3)	194 (90.7)	46 (93.9)	389 (96.3)	189 (94.5)	
**P/F ratio, m(IQR)**	267.5 [197.2,343.6]	300.6 [242.5,387.5]	104.0 [88.0,154.5]	256.7 [200.0,340.0]	272.0 [185.9,327.6]	<0.001
**S/F ratio, m(IQR)**	245.0 [200.0,250.0]	250.0 [242.5,250.0]	100.0 [94.8,106.6]	245.0 [227.5,250.0]	245.0 [198.0,250.0]	<0.001
**FiO_2_, m(IQR)**	0.4 [0.4,0.5]	0.4 [0.4,0.4]	1.0 [0.9,1.0]	0.4 [0.4,0.4]	0.4 [0.4,0.5]	<0.001
**PaO_2_, m(IQR)**	110.0 [87.0,141.0]	131.0 [99.0,159.0]	91.0 [75.0,108.0]	107.0 [85.0,136.0]	111.5 [87.0,138.0]	<0.001
**PaCO_2_, m(IQR)**	36.0 [31.0,41.0]	34.0 [30.0,39.0]	35.0 [30.0,45.0]	38.0 [33.5,43.0]	33.0 [29.0,37.0]	<0.001
**MAP, m(IQR)**	86.0 [78.0,96.0]	82.1 [75.0,89.9]	83.0 [77.5,92.0]	92.8 [85.0,103.1]	79.0 [74.0,86.0]	<0.001
**Creatinine, m(IQR)**	1.4 [0.9,2.8]	4.1 [1.7,6.9]	1.6 [1.1,3.1]	1.1 [0.8,1.6]	1.4 [0.8,2.4]	<0.001
**Bilirubin total, m(IQR)**	0.7 [0.5,1.4]	0.6 [0.4,1.1]	0.9 [0.5,2.5]	0.7 [0.5,1.2]	1.2 [0.6,3.7]	<0.001
**Albumin, m(IQR)**	3.0 [2.5,3.6]	2.9 [2.5,3.3]	3.0 [2.6,3.5]	3.5 [3.1,4.0]	2.2 [1.9,2.6]	<0.001
**Lactic acid, m(IQR)**	2.3 [1.7,3.7]	2.1 [1.6,3.2]	2.6 [1.9,4.3]	2.2 [1.7,3.3]	3.0 [2.0,5.0]	<0.001
**D-dimer, m(IQR)**	5220.0 [2041.0,15974.0]	5631.0 [2370.5,21648.0]	4898.0 [2668.0,10731.5]	3953.0 [1551.0,7646.0]	8468.0 [2529.4,22770.5]	<0.001
**Platelets, m(IQR)**	188.0 [119.0,260.5]	178.5 [110.8,255.5]	147.0 [97.0,235.0]	213.5 [150.8,276.2]	154.0 [83.5,232.5]	<0.001
**Hemoglobin, m(IQR)**	10.9 [8.7,12.9]	9.4 [7.9,11.4]	11.0 [8.9,12.7]	12.2 [10.9,14.0]	9.0 [7.7,10.4]	<0.001
**BNP, m(IQR)**	269.0 [105.0,873.5]	590.0 [247.0,1501.0]	222.0 [77.0,653.0]	213.0 [92.0,736.0]	215.5 [103.8,675.5]	<0.001
**BUN, m(IQR)**	27.5 [16.0,51.0]	62.0 [40.0,92.0]	34.0 [20.0,52.0]	19.5 [13.0,31.6]	23.0 [15.0,41.0]	<0.001
**SOFA max total, m(IQR)**	6.0 [4.0,9.0]	8.0 [6.0,10.0]	7.0 [5.0,10.0]	5.0 [3.0,7.0]	8.0 [5.0,10.0]	<0.001
**GCS total score, m(IQR)**	14.0 [11.0,15.0]	14.0 [10.0,15.0]	14.0 [12.0,15.0]	14.0 [10.0,15.0]	14.2 [12.0,15.0]	0.117
**PEEP, m(IQR)**	8.0 [5.0,8.0]	8.0 [5.0,8.0]	10.0 [8.0,10.0]	8.0 [5.0,8.0]	8.0 [5.0,8.0]	<0.001

For clinical variables, this table lists the medians and interquartile ranges (IQR: Q1-Q2) for each phenotype as well as for thewhole cohort. The p-value is also provided for each variable to indicate the statistical signifi cance of the differences among the phenotypes. For evaluating statistical signifi cance, Kruskal-Wallis test was performed for continuous variables and Chi-squared test was used for categorical variables.

* Mortality was computed with respect to patients (not encounters).

**Abbreviations used –** count: total encounters, mean: average, std: standard deviation, m: median, IQR: interquartile range, PaO_2_: partial pressure ofoxygen, SpO_2_: peripheral oxygen saturation level, FiO_2_: fraction of inspired oxygen, P/F: PaO_2_/FiO_2_ ratio, S/F: SpO_2_/FiO_2_ ratio,PaCO_2_: partial pressure of carbon dioxide in arterial blood, MAP: mean arterial blood pressure, Resp.: respiration, BNP: B-type natriuretic peptide, BUN: blood urea nitrogen, SOFA: sequential organ failure assessment, GCS: Glasgow coma scale.

**Measurement units –** P/F ratio, PaO2, PaCO2, and MAP: mmHg; S/F ratio and FiO_2_: unitless; creatinine and bilirubin total: mg/dL;albumin: g/L; lactic acid: mmol/L; D-dimer: ng/mL; platelets: ×10^3^/μL; hemoglobin: g/dL; BNP: pg/mL; BUN: mg/dL.

## Data Availability

Data and materials might be available upon request.

## References

[1] Singer M, Deutschman CS, Seymour CW, Shankar-Hari M, Annane D, Bauer M, The third international consensus definitions for sepsis and septic shock (sepsis-3). JAMA. 2016;315:801–10. 26903338 10.1001/jama.2016.0287 PMC4968574

[2] Coopersmith CM, De Backer D, Deutschman CS, Ferrer R, Lat I, Machado FR, Surviving sepsis campaign: research priorities for sepsis and septic shock. Intensive Care Med. 2018;44:1400–26. 29971592 10.1007/s00134-018-5175-z PMC7095388

[3] Parcha V, Kalra R, Bhatt SP, Berra L, Arora G, Arora P. Trends and geographic variation in acute respiratory failure and ARDS mortality in the United States. Chest. 2021;159:1460–72. 33393472 10.1016/j.chest.2020.10.042 PMC7581392

[4] Scala R, Heunks L. Highlights in acute respiratory failure. Eur. Respir. Rev. European Respiratory Society (ERS); 2018. p. 180008. 10.1183/16000617.0008-2018 PMC9489047 29592866

[5] Zampieri FG, Mazza B. Mechanical ventilation in sepsis. Shock. 2017;47:41–6. 27454388 10.1097/SHK.0000000000000702

[6] Grieco DL, Maggiore SM, Roca O, Spinelli E, Patel BK, Thille AW, Non-invasive ventilatory support and high-flow nasal oxygen as first-line treatment of acute hypoxemic respiratory failure and ARDS. Intensive Care Med. 2021;47:851–66. 34232336 10.1007/s00134-021-06459-2 PMC8261815

[7] Esteban A, Anzueto A, Frutos F, Alía I, Brochard L, Stewart TE, Characteristics and outcomes in adult patients receiving mechanical ventilation: a 28-day international study. JAMA. 2002;287:345–55. 11790214 10.1001/jama.287.3.345

[8] The acute respiratory distress syndrome, ” “The acute respiratory distress syndrome. The Journal of clinical investigation. 2012;122:2731–40. 22850883 10.1172/JCI60331 PMC3408735

[9] Gillespie DJ, Marsh HM, Divertie MB, Meadows JA 3rd. Clinical outcome of respiratory failure in patients requiring prolonged (greater than 24 hours) mechanical ventilation. Chest. 1986;90:364–9. 3743148 10.1378/chest.90.3.364

[10] Zhang Z. Multiple imputation with multivariate imputation by chained equation (MICE) package. Ann Transl Med. 2016;4:30. 26889483 10.3978/j.issn.2305-5839.2015.12.63 PMC4731595

[11] McInnes L, Healy J, Saul N, Großberger L. UMAP: Uniform Manifold Approximation and Projection. J Open Source Softw. 2018;3:861.

[12] M Lundberg SIL S.. A unified approach to interpreting model predictions. Advances in Neural Information Processing Systems 30. 2017. p. 4765–74.

[13] Sinha P, Delucchi KL, Chen Y, Zhuo H, Abbott J, Wang C, Latent class analysis-derived subphenotypes are generalisable to observational cohorts of acute respiratory distress syndrome: a prospective study. Thorax. 2022;77:13–21. 34253679 10.1136/thoraxjnl-2021-217158 PMC8688287

[14] Maddali MV, Churpek M, Pham T, Rezoagli E, Zhuo H, Zhao W, Validation and utility of ARDS subphenotypes identified by machine-learning models using clinical data: an observational, multicohort, retrospective analysis. Lancet Respir Med. 2022;10:367–77. 35026177 10.1016/S2213-2600(21)00461-6 PMC8976729

[15] Sinha P, Calfee CS. Phenotypes in acute respiratory distress syndrome. Curr Opin Crit Care. 2019;25:12–20. 30531367 10.1097/MCC.0000000000000571 PMC6814152

[16] Sinha P, Calfee CS, Delucchi KL. Practitioner’s guide to latent class analysis: Methodological considerations and common pitfalls. Crit Care Med. 2021;49:e63–79. 33165028 10.1097/CCM.0000000000004710 PMC7746621

[17] Cross M, Plunkett E. Kaplan Meier curves. Physics, Pharmacology and Physiology for Anaesthetists. Cambridge: Cambridge University Press; 2014. p. 376–376.

[18] Calfee CS, Delucchi K, Parsons PE, Thompson BT, Ware LB, Matthay MA, Subphenotypes in acute respiratory distress syndrome: latent class analysis of data from two randomised controlled trials. Lancet Respir Med. 2014;2:611–20. 24853585 10.1016/S2213-2600(14)70097-9 PMC4154544

[19] Aliberti S, Brambilla AM, Chalmers JD, Cilloniz C, Ramirez J, Bignamini A, Phenotyping community-acquired pneumonia according to the presence of acute respiratory failure and severe sepsis. Respir Res. 2014;15:27. 24593040 10.1186/1465-9921-15-27 PMC4015148

[20] Essay P, Mosier J, Subbian V. Rule-based cohort definitions for acute respiratory failure: Electronic phenotyping algorithm. JMIR Med Inform. 2020;8:e18402. 32293579 10.2196/18402 PMC7191347

[21] Essay P, Fisher JM, Mosier JM, Subbian V. Validation of an electronic phenotyping algorithm for patients with acute respiratory failure. Crit Care Explor. 2022;4:e0645. 35261979 10.1097/CCE.0000000000000645 PMC8893296

[22] Zhang Z, Zhang G, Goyal H, Mo L, Hong Y. Identification of subclasses of sepsis that showed different clinical outcomes and responses to amount of fluid resuscitation: a latent profile analysis. Crit Care. 2018;22:347. 30563548 10.1186/s13054-018-2279-3 PMC6299613

[23] Seymour CW, Kennedy JN, Wang S, Chang C-CH, Elliott CF, Xu Z, Derivation, validation, and potential treatment implications of novel clinical phenotypes for sepsis. JAMA. 2019;321:2003–17. 31104070 10.1001/jama.2019.5791 PMC6537818

[24] Aldewereld ZT, Zhang LA, Urbano A, Parker RS, Swigon D, Banerjee I, Identification of clinical phenotypes in septic patients presenting with hypotension or elevated lactate. Front Med (Lausanne). 2022;9:794423. 35665340 10.3389/fmed.2022.794423 PMC9160971

[25] Knox DB, Lanspa MJ, Kuttler KG, Brewer SC, Brown SM. Phenotypic clusters within sepsis-associated multiple organ dysfunction syndrome. Intensive Care Med. 2015;41:814–22. 25851384 10.1007/s00134-015-3764-7 PMC4607311

[26] Bhavani SV, Carey KA, Gilbert ER, Afshar M, Verhoef PA, Churpek MM. Identifying novel sepsis subphenotypes using temperature trajectories. Am J Respir Crit Care Med. 2019;200:327–35. 30789749 10.1164/rccm.201806-1197OC PMC6680307

[27] Pendergrass SA, Crawford DC. Using electronic health records to generate phenotypes for research. Curr Protoc Hum Genet. 2019;100:e80. 30516347 10.1002/cphg.80 PMC6318047

[28] He T, Belouali A, Patricoski J, Lehmann H, Ball R, Anagnostou V, Trends and opportunities in computable clinical phenotyping: A scoping review. J Biomed Inform. 2023;140:104335. 36933631 10.1016/j.jbi.2023.104335 PMC13246318

[29] Hripcsak G, Albers DJ. High-fidelity phenotyping: richness and freedom from bias. J Am Med Inform Assoc. 2018;25:289–94. 29040596 10.1093/jamia/ocx110 PMC7282504

[30] Shankar-Hari M, Rubenfeld GD. Population enrichment for critical care trials. Curr Opin Crit Care. 2019;25:489–97. 31335383 10.1097/MCC.0000000000000641

